# A Wrinkle in Measuring Time Use for Cognitive Health: How should We Measure Physical Activity, Sedentary Behaviour and Sleep?

**DOI:** 10.1177/15598276211031495

**Published:** 2021-07-28

**Authors:** Ryan S. Falck, Jennifer C. Davis, Karim M. Khan, Todd C. Handy, Teresa Liu-Ambrose

**Affiliations:** Aging, Mobility and Cognitive Neuroscience Laboratory, Department of Physical Therapy, Faculty of Medicine, 8166 University of British Columbia, Vancouver, BC, Canada(RSF, TLA); Djavad Mowafaghian Centre for Brain Health, 8166 University of British Columbia, Vancouver, BC, Canada(RSF, TLA); Center for Hip Health and Mobility, Vancouver Coastal Health Research Institute, Vancouver, Canada(RSF, KMK, TLA); Faculty of Management, 97950 University of British Columbia–Okanagan, Kelowna, BC, Canada(JCD); Department of Family Practice, Faculty of Medicine, 8166 University of British Columbia, Vancouver, BC, Canada(KMK); Attentional Neuroscience Laboratory, Department of Psychology, Faculty of Arts, 8166 University of British Columbia, Vancouver, BC, Canada(TCH)

**Keywords:** Physical activity, sleep, sedentary behaviour, cognitive health, measurement

## Abstract

One new case of dementia is detected every 4 seconds and no effective drug therapy exists. Effective behavioural strategies to promote healthy cognitive ageing are thus essential. Three behaviours related to cognitive health which we all engage in daily are physical activity, sedentary behaviour and sleep. These time-use activity behaviours are linked to cognitive health in a complex and dynamic relationship not yet fully elucidated. Understanding how each of these behaviours is related to each other and cognitive health will help determine the most practical and effective lifestyle strategies for promoting healthy cognitive ageing. In this review, we discuss methods and analytical approaches to best investigate how these time-use activity behaviours are related to cognitive health. We highlight four key recommendations for examining these relationships such that researchers should include measures which (1) are psychometrically appropriate; (2) can specifically answer the research question; (3) include objective and subjective estimates of the behaviour and (4) choose an analytical method for modelling the relationships of time-use activity behaviours with cognitive health which is appropriate for their research question.

## Introduction

Cognitive impairment and dementia overwhelmingly impact older adults.^1^ One new case of dementia is detected every 3 seconds.^2^ An estimated 10–20% of adults over 65 years of age are living with mild cognitive impairment,^3^ a transitional stage between healthy cognition and dementia,^4^ which is associated with up to a 30% increased risk of developing dementia within 5 years.^5^ As there is no pharmaceutical cure for cognitive decline and dementia readily available, quality research examining modifiable lifestyle factors that mitigate dementia risk is critical.^6^

Each moment of the day is spent in one of three basic activities which may play a critical role in maintaining older adult cognitive health – physical activity (PA), sedentary behaviour (SB) and sleep.^7-11^ Each of these three behaviours is independently associated with older adult cognitive health. There have been relatively few studies of the inter-relationship of these behaviours with cognitive health – most studies focus on one of them (sometimes two) – but the study of all three is at a relatively immature stage of research.

Given the complex interrelationships of PA, SB and sleep, and how they impact cognitive health, we wanted to critically examine the methods used to capture and analyse these behaviours associated with cognitive health. Measurement, analysis and interpretation of how these behaviours impact cognitive health are difficult. Physical activity is challenging to measure reliably in older adults.^12^ For example, self-reported older adult PA is open to reporting errors from anxiety, depression and cognitive impairments; objective estimates of PA can be inaccurate due to mobility impairments (eg slowed gait) or non-wear time. While there is now largely a consensus on the definition of SB,^13^ early research was plagued by different definitions of the concept.^10^ Sedentary behaviour in early epidemiological studies was classified as persons who did not report enough moderate or vigorous PA to be categorized as ‘active’, rather than on the basis of measured participation in SB (ie low energy activities spent sitting or lying down). This issue of different definitions of SB was compounded by variability and changes over time in the technology used to measure it.^14^ This lack of consensus in terminology has in part prevented an estimation of the magnitude of the relationship between SB and cognitive health.^11^ Human sleep is a complicated physiological and psychological phenomenon,^15^ with the physiological aspects of sleep being governed by homoeostatic and circadian processes (ie Process S and C, respectively) which interact continuously to regulate sleep.^16,17^

Reviews of the methods used to capture each of these behaviours suggest that the measurement of each time-use activity behaviour has been less than rigorous (eg measures lack evidence of validity and reliability, population specificity and/or sensitivity to change).^11,18,19^ We think that measuring each behaviour rigorously is important and that efforts should continue to improve PA, SB and sleep measurement.

However, time waits for no one, and researchers have identified that time-use activity behaviours are interrelated with each other and cognitive health.^9,20^ Rosenberger et al recently posited the *24-Hour Activity Cycle* model as a paradigm for exploring inter-relatedness of the health effects of SB, light PA, moderate-to-vigorous PA and sleep. There are also now guidelines for how much PA, SB and sleep is needed for health for children and adults of all ages.^21,22^ These guidelines broadly suggest that adults aged 18–64 years should (1) perform at least 150 min/week of moderate-to-vigorous PA and several h/week of light PA; (2) limit SB to 8 h/day or less including no more than 3 h/day of recreational screen time and (3) get 7–9 h/night of good-quality sleep on a regular basis with a consistent bed and wake-up time.^22^ The guidelines for adults over 65 years suggest that they get 7–8 hours/night of sleep but provide similar suggestions for PA and SB. Given the recent development of these guidelines, we should expect that researchers will be measuring multiple time-use activity behaviours in their studies more frequently, as well as examining how these behaviours interact with each other in order to impact cognitive health.

Measuring multiple time-use activity behaviours continuously only increases the complexity of a measurement protocol. Moreover, as we discuss within this article, there are specific measurement and analytical considerations for examining how time-use activity behaviours impact cognitive health. These considerations include (1) an understanding of the important psychometric properties for time-use activity behaviour measurement; (2) appreciation of what each time-use activity behaviour measurement tool can measure (and what it cannot); (3) the importance of including objective and subjective measures of each behaviour and (4) knowledge of the available analytic techniques for investigating the interrelationships of these behaviours. This article addresses each of these four considerations that researchers should be aware of when examining how PA, SB and sleep impact cognitive health.

## What Are Time-Use Activity Behaviours?

Each moment of our day is spent in one of three basic activities: PA, SB and sleep.^7^ Each of these behaviours is mutually exclusive, has different energy expenditures from each other^10,23,24^ and is physiologically distinct phenomena – both in the periphery and the brain.^25-31^

**Figure 1.**
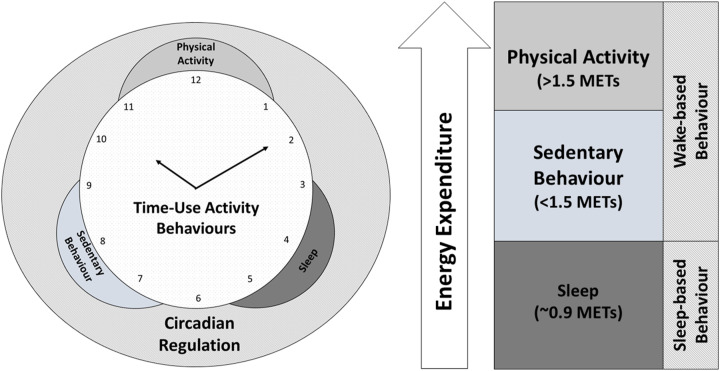
Time-use activity behaviours are distinct but related and are also part of circadian regulation. Abbreviation: METs, metabolic equivalents.

There are at least four separate terms used to collectively describe PA, SB and sleep. The current Canadian 24-hr guidelines^22^ refer to these as *movement behaviours*, where each behaviour reflects a certain amount (or lack) of movement. Bussman and Berg-Emons^32^ coined the term *physical behaviours* in 2013 to describe ‘the behaviour of a person in terms of body postures, movements, and/or daily activities in his/her own environment’. Pedišić et al^7^ collectively branded PA, SB and sleep as *time-use behaviours*. Rosenberger et al^20^ referred to these behaviours collectively as the *24-hr activity cycle*, which they calculated as being composed of four distinct activities: (1) SB; (2) light PA; (3) moderate-to-vigorous PA and (4) sleep. We broadly refer to PA, SB and sleep as *time-use activity behaviours* (Figure 1) since each 24-hour day is spent in some assortment of sleep, SB and PA; however, we think researchers need to a reach a consensus about the terminology to collectively define these behaviours.

Time-use activity behaviours are also linked to the circadian clock – the ∼24-hour biological clock that helps to align the sleep–wake cycle with the solar light–dark cycle.^33^ Specifically, time-use activity behaviours can be dichotomized into two broad and distinct types of behaviour which comprise the circadian cycle: (1) *wake-based behaviour* and (2) *sleep-based behaviour.* Wake-based behaviour occurs while a person is awake and out of bed and consists of both PA and SB. Sleep-based behaviour occurs only while sleeping. Together, these behaviours comprise both ends of the circadian cycle (ie sleep and wake). Time-use activity behaviours thus have a dynamic relationship with each other and with circadian regulation.

**Figure 2.**
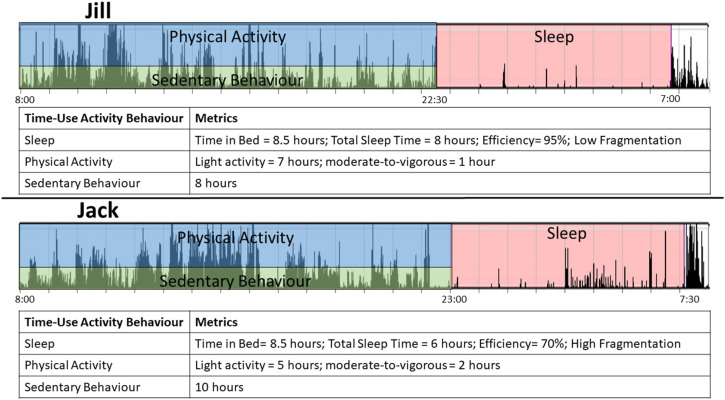
Example of two different time-use activity behaviour patterns. Jill engages in less SB, has more total PA and light PA and has better quality sleep than Jack; however, Jack spends more time in moderate-to-vigorous PA. Importantly, both Jill and Jack spend the same amount of time in bed each night, demonstrating that good sleep quality is not just based on duration but also efficiency and fragmentation, among other variables. Abbreviation: SB, sedentary behaviour; PA, physical activity.

To illustrate the complexity of time-use activity behaviours, we provide an example of wrist-worn accelerometry data for two separate older adults (we will call them Jack and Jill) with very different time-use activity behaviour patterns which are presented in Figure 2. Assuming that both Jack and Jill wake up at the same time (8:00), and Jill spends more time in PA and less time in SB than Jack; however, Jack spends more time in higher intensity PA than Jill but less time in lighter intensity PA. Jack and Jill also have very different sleep patterns even though they spend the same amount of time each night trying to sleep; Jack has less total sleep time, as well as less efficient and more fragmented sleep than Jill.

These differences in PA, SB and sleep between Jack and Jill may have vastly different implications on their cognitive health. For example, Jack’s poorer sleep quality and quantity may put him at higher risk for cognitive decline than Jill.^34^ Jill may be less at risk for cognitive decline and dementia than Jack because of her healthy sleep pattern, high amount of total PA^8^ and lower amount of SB.^11^

## The Challenges of Measuring Time-Use Activity Behaviours

Accurately measuring each time-use activity behaviour requires careful consideration of the psychometric properties of the tool used.^35^ These psychometric properties which are essential for measuring time-use activity behaviours include the need for validity, reliability, specificity and sensitivity to change.

The *validity* of the measure (ie the instrument) refers to the soundness of the interpretations of the results of a measurement by which accurate conclusions may be drawn from the results.^36^ No instrument can be ‘validated’, but rather different types of evidence of validity (ie definitional, content, criterion-based and construct validity) are collected to validate the interpretations made from an instrument.^37^ The *reliability* of an instrument refers to the degree to which measurements of the same trait are reproducible under the same conditions^38^ and is an important means by which an instrument can be deemed to have accuracy and consistency.^39^ As with validity, an instrument can have evidence of reliability (either norm-referenced or criterion-referenced), but this does not mean that the instrument is always ‘reliable’. *Population specificity* refers to the characteristics of the sample in which an instrument has evidence of validity and reliability. The specificity of a population can refer to a number of different characteristics such as age, ethnicity or sex. Using tools that do not have evidence of validity and reliability for the population they are presently measuring greatly reduces the ability to accurately interpret findings.^40^ Lastly, *sensitivity to change* (or responsiveness) refers to the ability of an instrument to detect change over time.^35^ Evidence of validity and reliability are requirements in order for the instrument to have sensitivity to changes over time, and change is typically quantified using the effect sizes for paired differences.

Are studies which measure time-use activity behaviours following these measurement principles? We suggest that measurement could be more rigorous than it has been. Beginning with PA, our group conducted a systematic review of PA intervention studies which found that only 63% of the measures used to estimate PA adhered to the principles of measurement.^41^ An appraisal of the current methods for measuring PA in people with neuromuscular disorders found that few of these measures have evidence of validity.^42^ Sattler et al^43^ recently conducted a systematic review of PA questionnaires; for many questionnaires, only one measurement property was established.

There is little evidence to suggest that SB and sleep are being measured more rigorously than PA. In a systematic review of studies examining the association between SB and cognitive function, half of the studies included in this review used a measure of SB that adhered to the principles of measurement.^11^ We are unaware of any study which has looked at measurement rigour in methods for measuring sleep. However, we looked at a recently published systematic review which examined the association between subjective sleep and cognitive performance^19^; seven of the 18 studies included in this review adhered to the principles of measurement (Supplementary Appendix).

## Physical Activity and its Measurement

**Figure 3.**
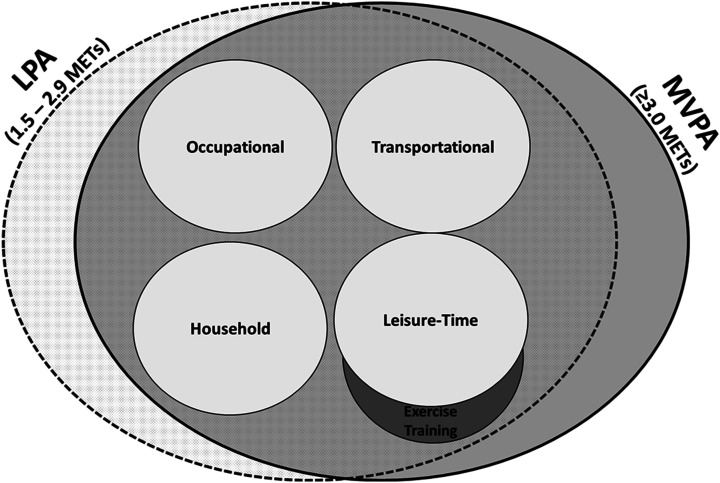
Physical activity domains and intensities. Abbreviation: LPA, light physical activity; MVPA, moderate-to-vigorous physical activity; METs, metabolic equivalents.

Physical activity is defined as a wake-based behaviour of any bodily movement produced by skeletal muscles which results in energy expenditure.^44^ Physical activity can be classified by its *duration* (ie time), the *frequency* that it occurs (usually in days/week), the energy expenditure required to perform it (*intensity*), the *type* of PA performed (eg cycling and walking) and the *context* (location as well as social setting – that is, alone versus in a group). The different types of PA can be broadly grouped into four basic domains in which PA occurs: (1) *occupational*, (2) *transportational*, (3) *household* and (4) *leisure time* (Figure 3).^45,46^ A sub-category of leisure-time PA is *exercise training*, which refers to planned, structured and repetitive bodily movement done to improve one or more components of physical fitness.^44^

Physical activity can also be classified by the energy expenditure which is required to perform it. *Light intensity PA* (LPA) involves activities wherein energy expenditure is at a level of 1.6–2.9 metabolic equivalents (METs) or 1.6–2.9 times an individual’s energy expenditure at rest.^10^ Light intensity PA includes activities such as slow walking, cooking food, washing dishes and standing still; it also includes activities done from a seated or lying position, which require 1.6–2.9 METs (eg crafts and stretching).^47^ *Moderate PA* consists of activities wherein energy expenditure is between 3.0 and 6.0 METs and *vigorous PA* consists of activities >6.0 METs. Evidence suggests that >150 min/week of PA of ≥3.0 METs has substantial benefits on numerous health outcomes,^28^ and thus, moderate and vigorous PA are generally examined as a single construct (ie *moderate-to-vigorous PA* (MVPA); ≥3.0 METs). LPA and MVPA are not exclusive to any one domain of PA, and thus, all domains of PA include activities which are LPA and MVPA.

**Figure 4.**
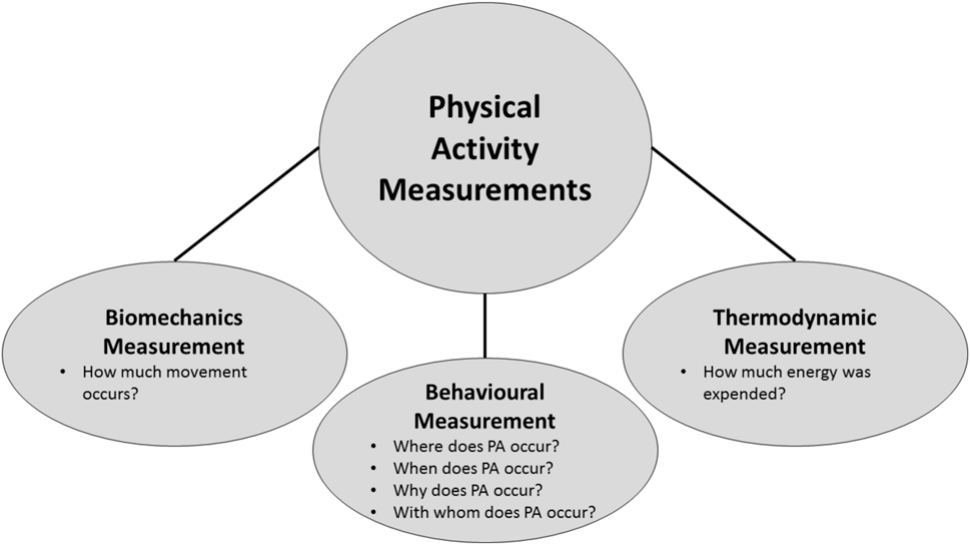
Dimensions of physical activity measurement.

There are three primary dimensions in which PA can be measured: *behavioural measurement* (ie the type and context of PA), *biomechanical measurement* (the movement of the body through space) and *thermodynamic measurement* (how much energy is expended) (Figure 4). Each of these dimensions of PA measurement is related; for example, 1 minute of walking has a different rate of force production generated by skeletal muscle and total energy expenditure than 1 minute of running. However, each of these dimensions has a different unit and scale of measurement. Using the previous example, walking can be measured quantitatively as a behaviour in minutes or distance travelled. The biomechanical measurement of walking could be in torque (ie force production by the muscle) or total work (force × distance); energy expenditure can be measured in METs, absolute oxygen consumption (L/min), relative oxygen consumption (ml/kg/min) or kcal. Accurately measuring each dimension of PA using a single measurement tool is thus not possible, and there is no single best method for measuring PA.

**Figure 5.**
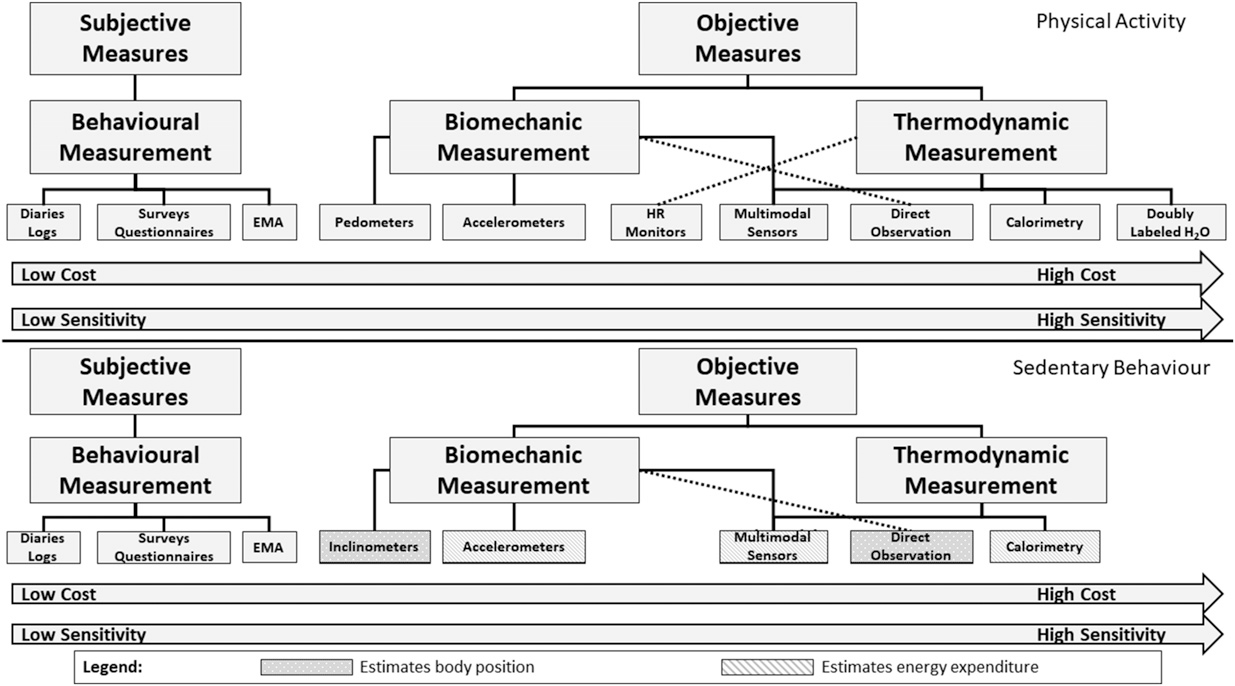
Characteristics and considerations for choosing a physical activity or sedentary behaviour measure. Top panel shows physical activity measures; bottom panel shows sedentary behaviour measures. Abbreviation: EMA, ecological momentary assessment.

The complex and multidimensional structure of PA has thus led to the development of a variety of methods for measuring that have been categorized in multiple ways.^40,48-50^ Physical activity measures can be broadly classified as either subjective or objective measures (Figure 5). *Subjective measures* assess a person’s subjective experience or recall of PA using questionnaires, surveys, diaries, logs or ecological momentary assessment. Subjective measures can provide useful information on usual (or past) PA behaviour – specifically, the amounts, types and contexts in which an individual engages in PA. *Objective measures* do not require the person being measured to recall or respond but instead use instruments (ie pedometers, accelerometers, heart rate monitors, multimodal sensors, calorimetry and doubly labelled water) or systematic observation to quantify an individual’s PA. Multimodal sensors refer to objective measures which use two or more objective instruments (eg accelerometry and heart rate) to quantify time-use activity behaviour.

There are benefits and disadvantages to subjective measures and objective measures. Subjective measures are inexpensive, easy to administer and commonly used in epidemiological and population-wide studies.^35^ These PA measurement methods provide important information about the context in which PA occurs (ie where, when and with whom); however, subjective PA measures are open to significant biases including social-desirability bias and recall bias.^51^ Recall bias is especially important for older adults since older adults are more likely to experience issues with accurately recalling their past or present PA.^12^ Subjective measures of PA also often fail to capture low-intensity activities (ie LPA).^52^ Other problems for subjective measures include issues with reliability, evidence of validity and sensitivity to change.^53^ Conversely, objective methods are often considered a more accurate and reliable measure of PA by eliminating recall bias; however, objective methods are more costly and require skilful administration and data interpretation.^54^ Objective methods can also lead to inaccurate estimates due to mobility impairments such as slowed gait (leading to false representations of PA) or non-wear time – either because the participant forgets to wear the device or because certain activities (eg swimming) cannot be measured with some objective measures.

## SB and its Measurement

Sedentary behaviour is defined as any wake-based behaviour which incurs ≤1.5 METs and includes behaviours such as sitting, television watching and lying down.^10^ A common misconception is SB is the inverse of PA; however, SB is an independent behaviour with its own distinctive effects on health.^55^ Like PA, SB can also be classified by its duration, frequency, type and context; SB is not classified by its intensity since all SB requires low energy expenditure. Tremblay et al^26^ proposed that SB should be classified according to the *SITT* formula, consisting of

1. SB frequency (number of bouts of a certain duration);
2. interruptions (eg getting up from the couch while watching television)^56^;
3. time (duration of sitting) and
4. type (mode of SB such as television viewing, driving a car or computer use).

**Figure 6.**
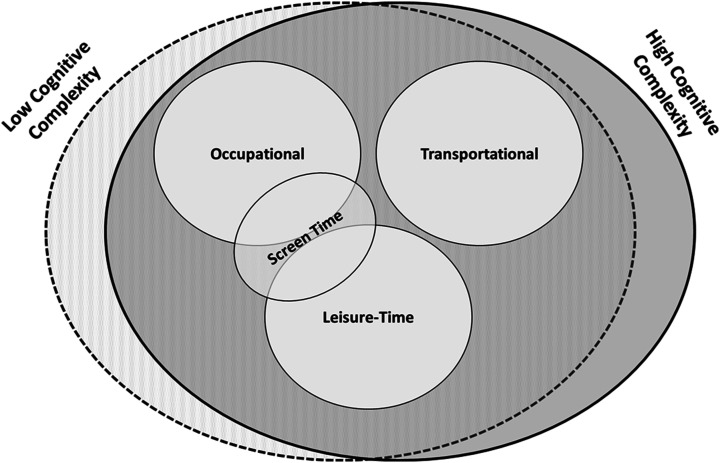
Types of sedentary behaviour.

Different types of SB can also be classified into three broad domains: (1) *occupational*, (2) *transportational* and (3) *leisure time* (Figure 6). There are also two important sub-categorizations of SB. *Screen time* refers to time spent watching television or using a computer and can be classified as either occupational or leisure-time SB. We propose that SB can be further classified based on the *cognitive complexity* required for the activity (eg low complexity versus high). This is particularly important for examining how SB impacts cognitive health since sedentary activities like computerized cognitive training can also promote cognitive health.^57^ Finally, the context of where SB occurs (and who with) can also have important implications on health.^58^

There are a few differences between measures of SB and measures of PA. Given that SB is a distinct and independent behaviour from PA, there are several objective measures of PA which cannot easily estimate SB (ie pedometers and heart rate monitors). Unlike the measurement of PA, wherein energy expenditure above a threshold of 1.5 METs is indicative of PA, SB refers to any activity which is performed from the seated or lying position while awake. Hence, the precise objective measurement of SB also requires information about *body position*, which can be detected through inclinometry.^10,26^ Not all SB measurement tools are capable of determining body position, and thus, these measures only estimate SB based on energy expenditure (either measured directly or indirectly estimated from accelerometer-based devices; Figure 5). However, there is no SB measurement tool currently capable of accurately estimating body position *and* energy expenditure continuously; measurement tools capable of estimating body position are thus only able to classify time spent sitting, lying down or standing.

## Sleep and its Measurement

Human sleep is a complex multidimensional phenomenon. Carskadon and Dement^59^ defined sleep as a recurring, reversible neurobehavioural state of relative perceptual disengagement from (and unresponsiveness to) the environment, which is typically accompanied in humans by postural recumbence, behavioural quiescence and closed eyes. The National Institute of Mental Health^60^ defined sleep and wakefulness as endogenous, recurring, behavioural states which are regulated by homoeostatic and circadian processes and reflect coordinated changes in the dynamic functional organization of the brain which optimizes physiology, behaviour and health. Both of these definitions emphasize that human sleep is multi-dimensional and can be measured across multiple levels of analysis.

**Figure 7.**
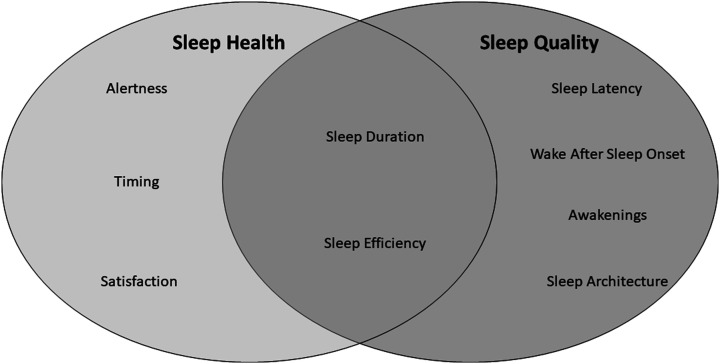
Classification systems for the different domains of sleep. The *sleep health* classification system classifies five different aspects of sleep which are related to health outcomes. *Sleep quality* is defined by six distinct aspects of sleep. These classification systems share similarities, but also classify different aspects of sleep as important.

Classifying the different aspects of sleep is challenging, and two distinct classification systems exist which share similarities and differences (Figure 7). First, empirical data demonstrate several characteristics of sleep which are related to health outcomes, and that can be measured using both objective and subjective methods.^61^ Broadly, these characteristics of *sleep health* are classified into five dimensions:

1. *Sleep duration*: The total amount of sleep obtained per 24 hours.
2. *Sleep efficiency/continuity*: The ease of falling asleep and returning to sleep.
3. *Timing*: The placement of sleep within the 24-hour day.
4. *Alertness/sleepiness*: The ability to maintain attentive wakefulness.
5. *Satisfaction/quality*: The subjective assessment of ‘good’ or ‘poor’ sleep.

Each of these dimensions is associated with health outcomes, although outcomes may differ by dimension. Importantly, each dimension can also be expressed in positive and negative terms – that is, there is directionality to what is considered ‘better’ and ‘worse’. For instance, the current 24-hour movement guidelines suggest that adults obtain between 7 and 9 hours of sleep each night^22^; sleeping less than 7 hours or more than 9 hours would thus be considered ‘worse’ sleep duration.

*Sleep quality* is another term widely used by researchers, clinicians and the public in reference to how well a person sleeps; however, the term sleep quality is vague and lacked definitional consensus until recently. The National Sleep Foundation recently defined several domains of sleep quality including (1) *sleep efficiency* (ie ratio of time spent sleeping to time spent trying to sleep); (2) *sleep latency* (length of time in minutes it takes to transition from wake to sleep); (3) *sleep duration* (total time spent sleeping); (4) *awakenings* (number of times a person wakes after imitating sleep); (5) *wake after sleep onset* (WASO; the time spent awake after sleep has been initiated and before final awakening) and (6) sleep architecture.^62^ Sleep quality can also be categorized as objective and subjective.^63^ *Objective sleep quality* can be classified according to the physiological aspects of sleep, while *subjective sleep quality* is based on how an individual feels about their sleep. Importantly, older adults’ objective sleep is poorly correlated to their subjective sleep,^15^ suggesting that objective and subjective sleep provide different information about older adult sleep.

**Figure 8.**
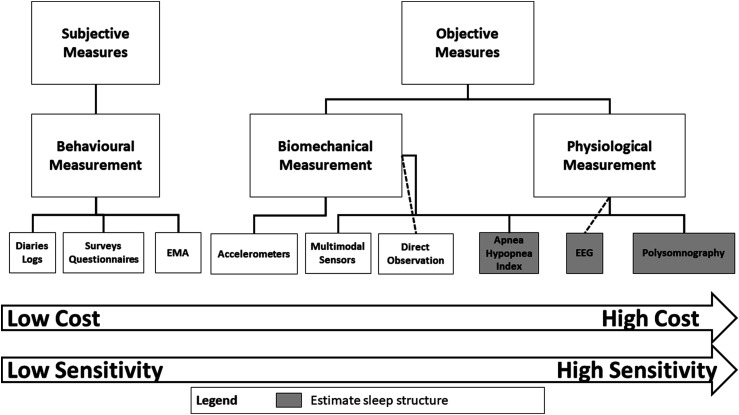
Characteristics and considerations for choosing a measure of sleep. Abbreviation: EMA, ecological momentary assessment.

Precise measurement of sleep in humans is thus challenging. As with PA and SB, sleep can be measured objectively and subjectively and using a wide variety of measures. Buysse^61^ highlighted that sleep can be characterized using self-report, behaviour, physiology (eg sleep architecture), neural circuits, cellular and genetic analyses. We acknowledge that sleep can be characterized in animals at the cellular and genetic levels^64,65^; however, the application of these measures for indexing human sleep is unclear. We therefore suggest that there are three broad domains by which sleep can be measured (Figure 8). *Physiological measurement* provides information about the distinct physiological processes, neural circuitry and architecture of sleep. Cellular and genetic measures of sleep could also be considered physiological measurement. *Biomechanic measurement* provides information about the movement of the body during sleep and can be used to estimate sleep health and quality. Subjective measures can be used for understanding the dimension of *behavioural measurement* – that is, the distinct behaviours, perceptions and characteristics of sleep health and sleep quality which are subjective in nature. Objective and subjective measures of sleep measure different components of sleep; hence, there is no best measure for assessing sleep.

The criterion measure for estimating physiological sleep is polysomnography (PSG).^66^ Polysomnography monitors a number of physiological processes while a subject is sleeping including brain activity (ie EEG), eye movements, muscle activity, heart rate, breathing functions (respiratory air flow and respiratory effort) and pulse oximetry. Polysomnography can provide information about sleep architecture, sleep-related breathing and other markers of sleep quality. While PSG thus provides accurate, reliable and sensitive information about a person’s sleep, PSG is expensive, time-consuming and requires significant participant and researcher burden. Indeed the invasive nature of PSG – usually requiring an overnight stay in a sleep laboratory or clinic – makes long-term multi-night recordings impractical.

Estimating sleep health and quality using wrist-worn accelerometry is an increasingly popular alternative for objectively measuring sleep, especially since these devices can be used to observe multiple days of sleep under normal daily-living conditions. Wrist-worn accelerometers have also been validated for measurement of sleep parameters by comparison with PSG,^67^ and thus, accelerometry is currently accepted as a valid, practical alternative to PSG, allowing for long-term continuous sleep assessments at home.^68,69^ While wrist-worn accelerometry does provide valid estimates of sleep, it is open to issues of validity and reliability compared to PSG – especially among individuals with chronic insomnia.^70^ Wrist-worn accelerometry also does not provide information about sleep architecture. Sleep can also be objectively estimated using multimodal sensors^71^; however, some of these devices (eg SenseWear Armband) may overestimate WASO, in part due to sleep processing algorithms.

Sleep can be measured using subjective methods such as diaries and questionnaires. Importantly, subjective measures of sleep likely measure different aspects of older adult sleep than objective measures.^15^ Subjective measures of sleep are quick and easy to administer and score and can discriminate ‘good’ versus ‘poor’ sleepers, but they are not able to detect subtle but clinically important changes in sleep quality due to age or disease.

## Measuring Time-Use Activity Behaviours Continuously

There is also an emerging body of evidence that PA, SB and sleep share a complex and dynamic relationship with each other and cognitive health.^8^ Both acute and chronic PA improve sleep quality,^72^ and the relationship between PA and sleep appears to be bidirectional.^73^ Wilckens et al^74^ determined that device-measured sleep efficiency mediates the relationship between PA and cognitive function in younger and older adults, while Lambaise et al^75^ observed that higher device-measured PA attenuated the consequences of poor sleep on the cognitive function of older women. Our research group found that PA and sleep have independent associations with older adult cognitive function, which suggests that PA and sleep might be related to cognitive health through multiple mechanisms.^76^ In a subsequent study, we determined that MVPA was associated with greater brain cortical thickness independent of SB.^77^ While these findings are mostly cross-sectional and preliminary, there is clearly a growing interest in examining how time-use activity behaviours are dynamically related with older adult cognitive health. We speculate this interest will continue to grow, given the recent publication of 24-hour movement guidelines.^21,22^

When would a researcher wish to examine multiple time-use activity behaviours? We have highlighted several examples in the preceding paragraph, but now provide one more example for clarity. Sedentary behaviour is associated with poorer sleep and poorer cognitive health,^11,78^ but it is unknown whether SB moderates the relationship between sleep and cognitive function. One way of examining this question would be to separately measure SB and sleep, in addition to testing cognitive function using a neuropsychological battery. For example, SB and sleep could be assessed separately by questionnaire, or separate devices could be worn by the participant to measure SB (eg an inclinometer) and sleep (eg PSG). The advantage of using separate measures is dependent upon the research design and the question of interest. In the present example, measuring SB and sleep by questionnaire may be practical when the sample size is extremely large, while measuring SB and sleep by different devices could be beneficial if the researchers are interested in specific aspects of these behaviours (eg sleep architecture or time spent sitting) which cannot be easily estimated by a single device.

A researcher might also choose to use a measurement tool which can continuously measure two or more time-use activity behaviours. Tools which can measure time-use activity behaviours continuously have obvious advantages. For example, it reduces researcher and participant burden by allowing the capture of multiple time-use activity behaviours from a single measurement tool. Measuring time-use activity behaviours continuously can also help researchers examine day-to-day fluctuations between different behaviours using cross-lagged analyses,^73^ wherein previous night’s sleep is used to predict the following day’s PA or SB, or vice versa.

There are a few common methods for measuring PA, SB and sleep continuously. Presently, these methods include accelerometry, multimodal sensors and systematic direct observation. Irrespective of the method used to capture all three time-use activity behaviours continuously, only limited aspects of each behaviour can be estimated. For example, none of these methods capture the subjective aspects of sleep, and thus, there is no single best measure for capturing all time-use behaviours continuously.

There are also several theoretical frameworks for how circadian misalignment may affect physical and cognitive health.^6,79^ For example, Landry and Liu-Ambrose^6^ suggested that circadian dysregulation may be a key driver of Alzheimer’s disease risk; the authors also suggested exercise training (ie PA) as a possible means of re-aligning the circadian clock. Nonetheless, the extent to which time-use activity behaviours can act as entraining factors (ie *zeitgebers*) for the biological clock is unclear. For instance, Buxton et al.^80^ found that exercise in the morning or afternoon did not have clear effects on circadian phase, while exercise in the evening (∼6 pm) caused phase advancement and nocturnal exercise (∼12 am) caused phase delays. Baehr et al^81^ also found that nocturnal exercise also caused phase delays in both younger and older adults. However, Youngstedt et al^82^ recently concluded that morning exercise (∼7 am) and afternoon exercise resulted in phase advancement, while evening exercise resulted in phase delays; the authors found no effect of nocturnal exercise on the circadian clock. These conflicting results highlight the lack of clarity about how PA affects the circadian clock. We are also unaware of any studies which have investigated how SB can alter circadian phase, and while sleep is closely tied to the circadian clock, it is dependent on both a homoeostatic process depending on sleep debt (ie Process S) and a process controlled by the circadian pacemaker (Process C), which are independent of each other.^17^

Thus, while we propose exploring the complex relationships of time-use activity behaviours and circadian rhythms with cognitive health is an interesting idea for future research, there is not a method of simultaneously field-measuring PA, SB and sleep as markers of circadian regulation.^20,83^ Wrist-worn actigraphy is the only field method which is capable of measuring all three time-use activity behaviours, while also providing indices of circadian regulation based on the rest-activity cycle.^84^ The actigraphy guidelines of Migueles et al^83^ highlighted that measuring circadian rhythmicity provided complementary information to time-use activity behaviour data; however, while the authors provided a detailed explanation of how time-use activity behaviour actigraphy data could be analysed, they did not suggest that these data could be used to estimate circadian rhythmicity which they viewed as a separate construct. Rosenberger et al also did not provide guidance on this issue.^20^ Given these current knowledge gaps and limitations of field measures, we suggest that there is not presently a method by which time-use activity behaviour epidemiologists can investigate the influence of these behaviours on circadian regulation in the natural environment.

## Novel Technologies for Measuring Time-Use Activity Behaviour

Several recently developed technologies now exist which may help to improve the measurement of time-use activity behaviours. One promising strategy is to combine heart rate monitoring with accelerometry. Heart rate monitoring can be used to estimate PA through heart rate variability (ie the variation in duration of time intervals between successive heart beats),^85^ and researchers have also proposed that heart rate variability can be used to stage sleep.^86-88^ Unfortunately, sleep staging from heart rate variability alone lacks precision.^89,90^ Combining heart rate variability with accelerometry data may help improve the accuracy of sleep measurement; however, the results to date do not suggest that combining these technologies improves sleep measurement.^91,92^

Interestingly, the latest generation of wearable devices are now capable of measuring cardiac activity via photoplethysmography optical sensors, wherein these sensors provide a supplement to accelerometry in the classification of sleep and wake. Preliminary investigations suggest that these devices provide comparable estimations of sleep versus wake to research-grade accelerometry devices.^93,94^ Other multimodal sensors, such as the SenseWear armband (BodyMedia), have some evidence of validity and reliability for measuring each time-use activity behaviour^95-97^ but have modest accuracy for measuring sleep beyond its duration.

Another possibility is to use skin temperature to track time-use activity behaviour. The thermo-regulatory system of the human body is aimed at maintaining a constant core temperature of ∼37^o^C. During intense PA, metabolic heat production can increase by 10 to 20-fold with respect to heat production at rest^98^; this heat is transferred mainly through the skin which can be used to measure PA duration and intensity.^99^ Recent work has also indicated that sleep and wake times can also be tracked closely through wrist-skin temperature monitoring.^100^ We are unaware of any studies which have yet to measure how time-use activity behaviours can impact cognitive health using this technology but hope this technology increases in popularity.

There has also been an explosion of commercially available PA and sleep trackers, which allow individuals to monitor their general health. These commercially available devices allow users to monitor their PA and sleep on a daily or moment-by-moment basis. While we think these devices could be particularly useful for understanding how time-use activity behaviours impact cognitive health, we have several concerns about the devices. First, the algorithms which these devices use to assess PA, SB and sleep parameters remain industry secrets. Second, the validity of these devices for monitoring time-use activity behaviour is limited, regardless of the device being tested.^101-103^ For instance, in a recent systematic review of Fitbit devices (perhaps the most popular commercial brand), Hagheyegh et al^104^ determined that these devices tended to overestimate sleep duration and efficiency with a medium-to-large effect size and underestimate WASO with a medium-to-large effect size – although the authors noted that the lack of accuracy in sleep duration and efficiency were only in reference to devices which did not measure heart rate (ie devices that did not use sleep staging).

Fortunately, recent evidence suggests that commercially available devices are improving in their ability to estimate time-use activity behaviour. Roberts et al^105^ used machine learning to train and test sleep–wake classifications for several multimodal (ie heart rate and accelerometry) consumer devices. The authors determined that these multimodal consumer wearables could be used to develop epoch-by-epoch models of sleep and wake which rival existing research grade devices. However, the accuracy of these devices for measuring free-living PA and SB is still limited,^106^ and thus, we suggest that researchers continue to use research-grade devices whenever possible.

A final measurement tool for indexing time-use activity behaviours which we think requires closer consideration is light exposure. The sleep–wake cycle is synchronized with the solar light–dark cycle through external stimuli (ie *zeitgebers*) by a process known as *entrainment*.^107^ Light is the principal entraining factor of the human biological clock and exerts its influence on light-sensitive receptors in the retina.^108^ Light exposure data are also used in the calculation of the rest-interval from accelerometry; that is, the time between when the wearer turns off their lights and tries to go to sleep, and the time when they get out of bed to start their day.^109^ The rest-interval is generally based on the initial time marking a period of prolonged cessation/onset of both movement and light. Light exposure is also an interesting contextual variable for determining where PA and SB occur (eg indoors versus outdoors). There are guidelines for the measurement of light^110,111^; however, the applicability of light metres for measuring individual light exposure over time – and in a field setting – is poor.^112^ Most wearable light metres cannot easily estimate different components of light,^113^ and these devices still lack a readily available technique to make simple discriminations between types of light which people may be exposed to on a daily basis, such as light versus dark or electrical light versus daylight. Indeed, the current evidence suggests that a simple and broad cut-off for discerning daylight from other light (>1000 lux is assumed to be daylight) performs as well as a criterion-referenced cut-off.^112^ We therefore suggest that while light sensors could provide useful information about time-use activity behaviours, the current technology needs improvement before it can be truly useful to the field.

## How Should We Analyse the Dynamic Relationships of Time-Use Activity Behaviour with Cognitive Health?

There is no single best approach for analysing the contribution of each time-use activity behaviour to cognitive health. Scientists and statisticians have long known that all models are inherently wrong, but some are useful.^114^ With this aphorism in mind, we highlight several of the key analytic frameworks by which researchers can examine how time-use activity behaviours are dynamically related to cognitive health.

The most common analytic approach in the health sciences is to use the General Linear Model (GLM), which is a useful and compact framework for comparing how several variables affect or are related to different variables.^115^ In its simplest form, GLM is described as:

Data = Model + Error

The GLM is the foundation for several statistical tests, including analysis of variance, analysis of covariance, linear regression and correlational analyses, and structural equation modelling. These statistical tests are common across the psychological and health sciences, and most researchers will be familiar with the assumptions of GLM (ie linearity, independence, normality and equivariance). One of the key benefits of GLM is that it provides a simple, easily interpretable and well-understood structure by which researchers can untangle how PA, SB and sleep impact older adult cognitive health. Since all models are wrong, it is impossible to obtain a ‘correct’ model by excessive elaboration, and thus, a simple but evocative model can provide far greater explanation to how time-use activity behaviours are related to cognitive health than an overelaborate model.

The GLM does have potential limitations for examining how time-use activity behaviours are related to cognitive health given that it (1) assumes linearity of relationships and (2) there is perfect multi-collinearity among the mutually exclusive categories of behaviour which comprise the 24-hour day, which causes the covariance structure of the data to be negatively biased.^116^ The issue of multi-collinearity does pose a problem when analysing the impact of time use across all three behaviours continuously (ie PA, SB and sleep duration). However, the issue of multi-collinearity becomes less relevant if researchers (1) only examine the impacts of one or (at most) two time-use activity behaviours on cognitive health or (2) when analysing all three behaviours continuously, examine variables of sleep other than its duration (eg efficiency and latency), or PA above or below a certain threshold (eg only examine MVPA).

Several other approaches are worth mentioning. One approach is isotemporal substitution analysis, which uses the GLM framework to simultaneously model the specific activity being performed and the specific activity being displaced in an equal time-exchange manner (eg the impact of exchanging 1 minute of PA for 1 minute of SB or vice versea).^117^ Pedišić et al^7,118^ argued for the use of compositional data analysis for examining the contributions of each time-use activity behaviour to health. In this approach, time-use activity behaviour is considered to be a composition matrix and can be analysed according to the clustering of time-use activity behaviours at different times throughout the 24-hour day. Using GLM, time-use composition (expressed as a set of log-ratios) can be modelled as independent variables and cognitive health as the dependent variable.^119^ The benefits of either of these approaches are that they can be useful for examining how time-use allocation impacts cognitive health. For instance, isotemporal substitution can determine whether substituting PA for time spent in SB improves cognitive function or vice versa. Compositional data analysis can help determine how allocation of behaviours across the 24-hour day is associated with cognitive health. However, each time-use activity behaviour is complex and multi-dimensional. Time-use allocation is not the only factor which must be considered in the analyses of these behaviours. For example, it is unlikely that 9 hours of poor sleep (ie low sleep efficiency and minimal stage 3 and 4 sleep) is somehow better for cognitive health than 7 hours of high quality sleep. Isotemporal substitution and compositional data analysis are also open to other issues, including multi-collinearity and non-linearity.^117,119^

Given the limitations of GLM, Bayesian estimation methods (such as dynamic structural equation modelling) and non-parametric regressions are two other techniques which have utility for understanding how time-use activity behaviours impact cognitive health. One of the key advantages of Bayesian estimation over frequentist methods (eg GLM) is that it allows researchers to incorporate background knowledge (ie priors) into their analyses instead of essentially testing the same null hypothesis repeatedly without incorporating the lessons of previous studies.^120^ Frequentist methods assume a parameter is unknown, but fixed – that is, for a population, there is only one true mean or one true regression coefficient. In the Bayesian view, all unknown parameters are treated as uncertain and are therefore described by a probability distribution. This approach can be more intuitive because the focus of Bayesian estimation is on predictive accuracy rather than ‘yes or no’ significance testing. Bayesian methods also eliminate concerns about small sample sizes or handling non-normal parameters and can also be useful in the analysis of cross-lagged data (eg does previous night’s sleep predict following day’s PA and vice versa?).^73^ While Bayesian methods do present several benefits, priors included in a model might be chosen for opportune reasons such that results can be biased to one’s hypotheses. Bayesian statistical methods also assume that every parameter has a distribution in the population; frequentist methods do not make this assumption.^121^ This issue could be problematic for some cognitive markers; for instance, we would not expect that healthy younger adults would have any parametric distribution for white matter hyperintensities – a marker for vascular cognitive impairment which becomes apparent in older adulthood.^122^

Non-parametric regression analyses (eg regression splines) are an appealing method for the analysis of non-parametric estimations of the relationship of time-use activity behaviours and cognitive health. The benefit of this approach is that the relationship between parameters is not assumed to be linear a priori, and the relationship can instead be ‘curved’ to appropriately fit the data.^123^ This is especially useful when the relationship between variables is assumed to be non-linear. We think this approach can be beneficial for modelling the relationship between time-use activity behaviours and cognitive health, given that (1) cognitive health changes with age in a non-linear fashion, beginning in approximately the 3rd decade of life^124^; (2) the rate of cognitive decline is not linear^125^ and (3) time-use activity behaviours change non-linearly with age.^126,127^ While we think there is some potential for non-parametric analyses, the interpretations of results from these methods are often complex, and a large sample size is necessary in order to model non-parametric relationships.

Finally, we note that our discussion of this topic is not exhaustive. Migueles et al^83^ recently described the available analytical techniques for assessing time-use activity behaviour using accelerometry. Of particular interest, the authors developed a decision tree for determining the type of statistical analysis to be conducted using accelerometry data. Rosenberger et al^20^ also provided a broad description of the available methods for analysing time-use activity behaviours. We thus refer interested readers to these reviews for more information.

## Recommendations for Measuring and Analysing the Dynamic Relationships of Time-Use Activity Behaviours with Cognitive Health

The precise measurement of PA, SB and sleep each present unique challenges for researchers to consider when designing a measurement protocol. Measuring these behaviours continuously presents an added layer of complexity, although there are an increasing number of tools available which can estimate all three time-use activity behaviours continuously. However, researchers interested in how time-use activity behaviours are dynamically related to older adult cognitive health must carefully contemplate on the measures they plan to use, as well as how they wish to examine these relationships. We highlight below four key recommendations for examining the relationships of PA, SB and sleep with cognitive health.

### Use measures with evidence of validity and reliability, population specificity and adequate sensitivity for measuring the behaviour(s) of interest

Failing to use measures which have clearly documented psychometric properties can lead to inaccurate conclusions about how each time-use activity behaviour is related to cognitive health.^11,41^ For instance, using a questionnaire for estimating sleep quality which has evidence of validity in children, but not older adults, is likely to lead to inaccurate conclusions about how sleep quality impacts older adult cognitive health. We thus cannot overstate that the importance of using measures which have evidence of validity and reliability and population specificity for measuring the behaviour(s) of interest. Moreover, if the researcher is interested in observing changes over time, it is critical to choose a measure which has adequate sensitivity to change.

### Use measures which can specifically answer the research questions of interest

We think it is especially critical to emphasize the importance of construct validity when choosing a measure, given that each time-use activity behaviour is a complex and multi-dimensional construct. No measurement tool can capture all aspects of each behaviour. The research questions which are possible are predicated on the type of measurement tool(s) used. For example, if a researcher is interested in how subjective sleep quality impacts cognitive health, then using a subjective measure of sleep is likely to be more useful to answering the research question than objectively measured sleep (eg PSG or actigraphy). The measurement tools used will also dictate the research questions which are possible. For instance, using actigraphy or multimodal sensors can allow researchers to answer questions about the dynamic relationships of PA, SB and physiological sleep with cognitive health; however, questions about subjective sleep will require an additional measure of subjective sleep quality. We do not suggest that researchers need to choose measures which are capable of indexing multiple time-use activity behaviours – some research questions may only be interested in how one behaviour impacts cognitive health – but we do think that researchers should be aware of what they are trying to measure, and what they are not.

### Depending on the research question, aim to include objective and subjective measures for each time-use activity behaviour of interest

Objective and subjective measures provide complementary data about each time-use activity behaviour. For example, researchers interested in the interrelationships of older adult PA and sleep with cognitive health will likely be interested in how PA is associated with both subjective and objective sleep quality; the context in which PA occurs (eg morning or evening or indoors versus outdoors), which is typically measured by questionnaire, might also moderate the association between sleep and older adult cognitive health. In addition to the intensity and volume of PA, the context (eg playing with grandchildren and group-based exercise classes) and type of PA which older adults perform may be related to cognitive health. Some types of SB may be beneficial for cognitive function (eg cognitive training). Sleep is a multi-dimensional construct with both subjective and objective aspects that each has independent relationships with cognitive health. Given that sleep- and wake-based behaviour is closely tied to circadian regulation, we also think that the timing of when PA, SB and sleep occur should be measured more frequently.

### Researchers should choose an analytical method which can answer their research question and be aware of its benefits and limitations

Since all statistical models are inherently wrong,^114^ we think that good science requires researchers to choose analytical approaches which can answer their research question appropriately, while also being aware of what the limitations of their analyses are. Many questions can and probably should be answered using GLM since it provides a simple, easily interpretable and well-understood structure by which researchers can untangle how PA, SB and sleep impact older adult cognitive health. For example, an analysis of whether PA moderates the association between sleep and cognitive performance can likely be accomplished using multiple linear regression. However, we also recognize that isotemporal substitution analysis, compositional data analysis, Bayesian methods, non-parametric methods or other approaches we have not discussed may be suitable for answering certain research questions. Statistical analysis which is artfully employed can enable the researcher to deduce the validity of her hypotheses and will expand our knowledge about how time-use activity behaviours impact cognitive health.

## Concluding Remarks

Understanding the specific impacts and dynamic relationships of PA, SB and sleep with older adult cognitive health will be critical for identifying and developing more effective lifestyle strategies to promote healthy cognitive aging. Understanding these relationships will require well-designed measurement and analysis protocols to elucidate how these time-use activity behaviours are related to each other and cognitive health. It is critical that future studies aiming to examine the dynamic relationships of PA, SB and sleep adhere to the principles of measurement and carefully consider the research questions which they aim to answer.

## Supplemental Material

sj-pdf-1-ajl-10.1177_15598276211031495 – Supplemental Material for A Wrinkle in Measuring Time Use for Cognitive Health: How should We Measure Physical Activity, Sedentary Behaviour and Sleep? Click here for additional data file. Supplemental Material, sj-pdf-1-ajl-10.1177_15598276211031495 for A Wrinkle in Measuring Time Use for Cognitive Health: How should We Measure Physical Activity, Sedentary Behaviour and Sleep? by Ryan S. Falck, Jennifer C. Davis, Karim M. Khan, Todd C. Handy and Teresa Liu-Ambrose in American Journal of Lifestyle Medicine
