# Supplementary material for: A Wrinkle in Measuring Time Use for Cognitive Health: How should We Measure Physical Activity, Sedentary Behaviour and Sleep?
Source: Am J Lifestyle Med. 2021 Jul 28;17(2):258–75. doi: 10.1177/15598276211031495 (PMC9989499; doi:10.1177/15598276211031495)
Supplement: sj-pdf-1-ajl-10.1177_15598276211031495 – Supplemental Material for A Wrinkle in Measuring Time Use for Cognitive Health: How should We Measure Physical Activity, Sedentary Behaviour and Sleep? [file sj-pdf-1-ajl-10.1177_15598276211031495.pdf]

Appendix A. Evaluation of the psychometric properties of sleep measures from studies included in the systematic review and meta-analysis by Lo and colleagues (2016).

Study	Psychometric properties followed?
Auyeung et al., 2013	+
Benito-Leon et al., 2009	-
Devore et al., 2014	+
Faubel et al., 2009	-
Ferrie et al., 2011	-
Gildner et al., 2014	-
Keage et al., 2012	-
Kronholm et al., 2009	-
Lambaise et al., 2014	+
Lo et al., 2014	+
Loerbroks et al., 2010	-
Potvin et al., 2012	+
Ramos et al., 2013	-
Martin et al., 2012	+
Schmutte et al., 2007	-
Tworoger et al., 2006	-
Virta et al., 2013	-
Xu et al., 2011	+

+ Studies wherein the measure of sleep had evidence of validity and reliability, population specificity, and sensitivity to change.

- Studies wherein the measure of sleep was missing at least one psychometric property
